# University Students’ Behaviour towards Entrepreneurial Intention in Ecuador: Testing for the Influence of Gender

**DOI:** 10.3390/ijerph17228475

**Published:** 2020-11-16

**Authors:** Pablo Rodriguez-Gutierrez, Luis Javier Cabeza-Ramírez, Guzmán Antonio Muñoz-Fernández

**Affiliations:** Department of Business Organization, Agrifood Campus of International Excellence, ceiA3, Faculty of Law and Economic and Business Sciences, University of Córdoba, Pl./Puerta Nueva, s/n. 14002 Córdoba, Spain; pablo.rodriguez@uco.es (P.R.-G.); guzman.munoz@uco.es (G.A.M.-F.)

**Keywords:** countries in transition, entrepreneurial intention, efficiency-driven economies, university students, motivation, psychosocial factors

## Abstract

While the current global context of successive economic and health crises are punishing the economies of different countries in the world, it is particularly relevant to explore the business intentions of young university students, as potential entrepreneurs of opportunity. This matter is of the utmost importance, as it helps to facilitate the implementation of measures that can ensure the future recovery of the economy and the creation of new businesses. The objective of this paper is to study the institutional and psychological antecedents of entrepreneurial intention and the role of gender. The theory of planned behaviour is applied to assess how personal attitudes, subjective norms, and perceived behavioural control can affect students′ intention of becoming an entrepreneur. In addition, organizational support and institutional barriers are tested as potentially significant antecedents of entrepreneurial intention, along with the influence of gender. The research carried out was based on survey responses from a sample of 740 students of economics, communications, and education at an Ecuadorian university. The research propositions were tested using a partial least squares approach. Results indicate that behaviour towards entrepreneurship does not change in relation to gender. In addition, personal attitudes and perceived behavioural control regarding entrepreneurship are positively related to students′ entrepreneurial intention. Organizational support is also found to be important for generating entrepreneurial intention. The paper adds to the current knowledge base on entrepreneurial intention by analysing the individual and joint influence of the principal elements of the theory of planned behaviour, as well as organizational support and institutional barriers on entrepreneurial intentions. Moreover, the research provides a useful perspective on the antecedents of entrepreneurial intention in an unexplored context such as Ecuador, by responding to the call focusing on entrepreneurial intention in different regions, cultures, and contexts.

## 1. Introduction

There is a broad consensus on the multiple benefits of entrepreneurship and the start-up of new businesses; they include economic development, job creation, increased productivity, innovation, dynamic growth of the economy, and social well-being [1,2]. Developed and somewhat less developed countries thus promote entrepreneurship as a formula for securing these advantages [3,4]. In this context, the university has taken on a central role as a driver of opportunity entrepreneurship and a source of economic development for the development of young people [5,6,7]. The entrepreneurial spirit is seen as intentional behaviour. As such, there has been a growing amount of research in recent years on the cognitive factors shaping the motivations and desires that compel certain people to start their own business [8,9]. Within this body of research, various studies focus on the early stage in which university students—potential opportunity entrepreneurs [5]—forge their possible future entrepreneurial intention (EI) [10].

In the sphere of university education and EI, the research has primarily focused on the analysis of said intention in developed regions or economic environments [10]. For example, there have been studies of the EI of Spanish, Taiwanese, Finnish, Swedish, and Dutch students [11,12,13]; more occasionally [14,15]**,** the focus has been on less favourable contexts, centring on factors of production or so-called efficiency-driven economies [5]. Within the latter group, Ecuador is noteworthy for some of its specific features. According to Bosma, Hill, Ionescu-Somers, Kelley, Levie, and Tarnawa [5] and Lasio et al. [16], the perceptions and attitudes of its population as well its score on the entrepreneurial spirit index (GESI) point to a strong tradition of entrepreneurship, with societal values that support it. In addition, the country is notable for being an efficiency-driven economy with one of the highest rates of total early-stage entrepreneurial activity (TEA) and with virtually no gender gap [5,16] in terms of entrepreneurship. These features can also be observed among the younger university population [17].

In the current global context of successive economic and health crises, it is particularly relevant to explore the EI of young university students, potential opportunity entrepreneurs. This issue is of paramount importance, since it helps to facilitate the implementation of measures that can ensure the future revival of the economy and the creation of new businesses. The present study responds to the call for further research made by Liñán and Chen [12] and Maresch et al. [18] among others, focusing on EI in different regions, cultures, and contexts. Justified by the characteristics of the Ecuadorian economy described above, this paper seeks to address said knowledge gap. To do so, it uses a widely applied theory in analyses of intention [10]: the theory of planned behaviour (TPB) [19]. Therefore, the main aim of this work is to analyse the influence of the three antecedents of intention included in the TPB (personal attitude, PA; subjective norm, SN; and perceived behavioural control, PBC) on the EI of Ecuadorian students. In addition, following Ye et al. [20], the paper examines the influence of organizational support (OS) and institutional barriers (IB) on those elements of the TPB, accounting for the characteristic Ecuadorian cultural traits described above. The article makes a number of contributions to the research on EI in the academic field: it provides a more in-depth understanding of the role played by the aforementioned variables as well as the potential moderating influence of gender in the context of an efficiency-driven economy.

The rest of the article is structured as follows. Section 2 reviews the literature, sets out the hypotheses, and presents the research model. Section 3 addresses methodological issues, the measurement instrument and the sample. Section 4 reports the main empirical results. Section 5 presents a discussion of the results. Lastly, Section 6 outlines the conclusions, practical implications, limitations, and future lines of research.

## 2. Literature Review

Under the TPB approach, the shared values of culture affect the antecedents of intention [12] (p. 598). Countries′ entrepreneurial activity occurs in contexts with diverse social norms; social culture is slow to change, and traditions remain relatively stable despite globalization [21]. Nevertheless, as pointed out by Liñán, Nabi, and Krueger [13] and Bruton et al. [22], theories and analyses established in developed nations are expected to be applicable in other contexts such as emerging and efficiency-driven economies. The scope of the TPB may therefore be limited. Thus, Lortie and Castogiovanni [10] underscore the need to incorporate additional variables that contribute to a better understanding of the resulting EI. In this regard, the present study follows earlier work such as that by Urban and Chantson [23] or Feola et al. [24] in accounting for the effect of exogenous factors related to the cultural context: in this case, the focus is on IB and OS, as well as whether gender exerts a moderating effect in regions with equal representation in entrepreneurship.

### 2.1. Theory of Planned Behaviour

The sociocognitive approach proposed in the TPB is an extension of the theory of reasoned action (TRA) [25] developed by Fishbein and Ajzen [26]. The original theory holds that an individual′s intention to perform a behaviour is determined by two components. The first is his/her attitude towards it (PA), that is, the individual′s personal feelings—positive or negative—regarding the behaviour (if an individual has a favourable attitude his/her intention to perform the behaviour will be positive, and vice versa). The second is the subjective norm (SN), understood as the perception that the individual′s referents (family, friends, etc.) have of the behaviour (if an individual perceives that his/her referents approve of the behaviour, he/she will have a positive intention to do it, and vice versa). However, the individual′s behaviour may largely depend on volitional control, that is, his/her ability to perform the behaviour [19]. To overcome this limitation of the original theory, the concept of perceived behavioural control (PBC) [19] was introduced. This element is understood as the individual′s control over the necessary external resources, for example, knowledge, skills, or time (if an individual feels that he/she controls the resources needed to perform the behaviour, his/her intention towards it will be positive, and vice versa [19]). Therefore, the TPB postulates that the three abovementioned antecedents have an impact on intention, which in turn, is the strongest predictor of future behaviour. In this regard, the dependent variable, EI, is understood in an explicit sense as the intention to start a new business or create a new company [27]. Ferreira et al. [28] identify other paths to entrepreneurship, including self-employment or continuing a professional career, but they lie beyond the scope of this study.

In the area of entrepreneurship research, the TPB has become established as one of the most robust approaches for explaining EI [10,29]. It has been applied in different regions, countries, and cultural contexts [15,23,30], with factor- and efficiency-driven economies being exceptional case studies [14,15]. Accordingly, the three basic hypotheses of the TPB for an efficiency-driven economy such as the Ecuadorian one can be formulated as follows:

#### 2.1.1. Attitude towards Entrepreneurship

The dimension of attitude towards the act is a psychological construct that influences and predicts personal behaviours [31]. In this study, it refers to the degree to which the individual makes a favourable/unfavourable personal assessment of entrepreneurship [19]. From this perspective, entrepreneurial behaviour is understood as a consequence of prior attitudes rather than a spontaneous activity. There is a general consensus in the literature about the positive relationship between attitude and EI [32,33]. Ruizalba-Robledo et al. [34] found evidence of such a relationship among potential entrepreneurs in the university context.

**Hypothesis 1a** **(H1a).** *PA is positively related to EI.*

#### 2.1.2. Subjective Norm

The SN refers to the intensity with which intentionality is embedded in cultural expectations [35]. In this case, there is greater discrepancy about its effect on EI. On the one hand, Lima et al. [36] identified a positive influence on EI in a university setting; on the other, Ruizalba-Robledo, Vallespín-Arán, Martín-Sánchez, and Rodríguez-Molina [34] did not find any relationship between students′ SN and EI.

**Hypothesis 1b** **(H1b).** *SN is positively related to EI.*

#### 2.1.3. Perceived Control over Entrepreneurship

Perceived control over behaviour (or self-efficacy) refers to the individual’s perception of whether or not they have access to the resources and opportunities needed to carry out a specific task, particularly when the activity is new and challenging [37,38]. In this regard, subjects with a stronger sense of self-efficacy can see more opportunities in a risky choice and may even take more risks than others. It is widely agreed in the literature that self-efficacy has a positive effect on EI [36,39,40]. In the university context, Krueger and Carsrud [41] and Ruizalba-Robledo, Vallespín-Arán, Martín-Sánchez, and Rodríguez-Molina [34] found a positive relationship between perceived control over entrepreneurship and the probability of becoming an entrepreneur.

**Hypothesis 1c** **(H1c).** *PBC is positively related to EI.*

### 2.2. Institutional Antecedents of EI

This study addresses the role played by the institutional environment or framework (e.g., [42,43]) in which students develop their behaviour towards entrepreneurship. Under this theoretical approach, students′ behaviour, as viewed through the TPB, is determined by the societal support that underpins these individuals’ vision of their professional future. Thus, two levels of antecedents that may shape students′ behaviour towards entrepreneurship can be identified: the context of the educational organization and the national socioeconomic context [23].

#### 2.2.1. Organizational Support (OS) as an Antecedent of Attitude and Perceived Control

OS includes the organizational culture in which students are immersed in and around the university [44]. One factor in OS is entrepreneurship education, which teaches the specific skills needed to take on the challenges faced by entrepreneurs [45]. OS also functions through the interactions, networks, and alliances arising among academics, students, and established companies [46].

Several university-level studies point to the impact of OS [23] on the EI of students and academics. A favourable institutional context can influence EI directly or indirectly through changes in individuals′ attitudes [41,44], and support from the institutions is expected to exert a positive influence on the individual′s PBC. For example, the study by Turker and Sonmez Selcuk [44] indicates that university students’ EI is related to the educational, relational, and structural support they receive. In the same vein, Urban and Chantson [23] argue that university policies and incentives become an indirect filter of start-up intentions through PA and PBC, citing Clarysse et al. [47] and Guerrero et al. [48], among other studies. Therefore, OS is considered an indirect antecedent of EI, giving rise to the following hypotheses:

**Hypothesis 2a** **(H2a).** *OS is positively related to PA.*

**Hypothesis 2b** **(H2b).** *OS is positively related to PBC.*

#### 2.2.2. Institutional Barriers (IB) as an Antecedent of Attitude, Subjective Norm, and Perceived Behavioural Control

There are many barriers that can act as a brake on students′ EI. Sandhu et al. [49] argue that the most common barriers in the stages prior to engaging in entrepreneurship are psychological: an aversion to risk, failure, stress, or the idea of hard work. However, this study focuses on university students′ perceptions of IB in their region or environment, e.g., lack of resources, government assistance or regulation, barriers to market entry, and even barriers related to knowledge [23,50]. In this regard, the perception of IB, and even the barriers that actually exist, can vary considerably depending on the level of development of the country. As Sandhu et al. [49] point out, developed countries provide more institutional support or have more advanced educational systems, which notably reduce these barriers.

**Figure 1:**
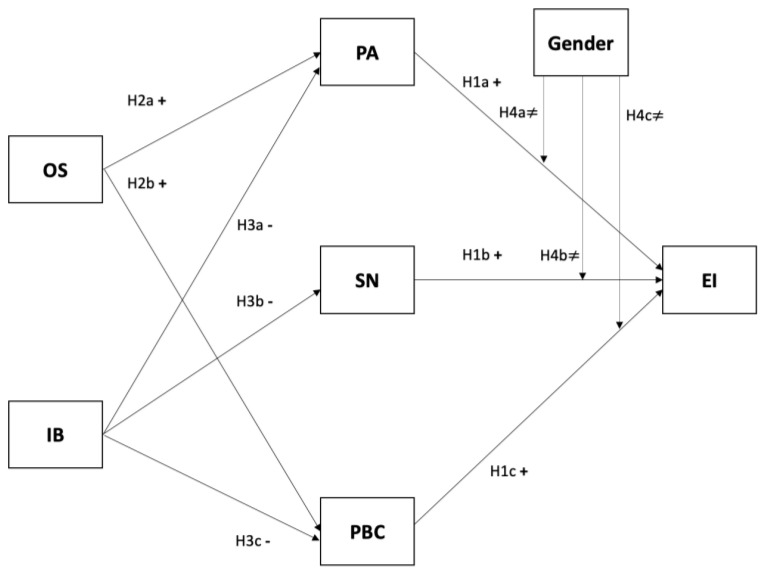
Research model and hypotheses.

Institutional factors can also constitute an incentive and form part of the country′s context or type of economy (factor-, efficiency-, or innovation-driven). According to Krueger and Carsrud [41], they are elements that can exert a direct effect on EI, or an indirect effect by influencing the individual′s PA towards entrepreneurship. In their analysis of EI in academics, Urban and Chantson [23] identify IB as an antecedent of intention that influences PCB. Other authors such as Yeganegi et al. [51] also raise this possibility. In this regard, Lasio, Ordeñana, Caicedo, Samaniego, and Izquierdo [16] define the Ecuadorian context as an efficiency-driven economy that scores very low on assessments of government policies and access to finance. It could therefore be argued that perceptions of IB would have a negative effect on the three antecedents of TPB. Thus, the next hypotheses that are summarized in the model in Figure 1 are formulated as follows:

**Hypothesis 3a** **(H1a).** *Perceptions of IB negatively influence EI through PA.*

**Hypothesis 3b** **(H3b).** *Perceptions of IB negatively influence EI through SN.*

**Hypothesis 3c** **(H3c).** *Perceptions of IB negatively influence EI through PBC.*

### 2.3. The Moderating Effect of Gender on EI in the University Context

Gender is commonly included in entrepreneurship research given its relevance in the formation of EI. Traditionally, women′s entrepreneurial spirit has tended to lie somewhat behind that of men [38,41]. Indeed, a number of different studies claim that women have different behavioural patterns from men, as a result of which they show less of a tendency to engage in entrepreneurship [52,53]. Rooted in liberal feminist theory, some research focuses on the environmental reasons that keep women at a disadvantage in this regard, such as women′s access to capital [54]. Other authors (e.g., [55]) highlight their tendency to take fewer risks or seek a better balance between work and family life, which may lead some to believe that the female gender role is not compatible with entrepreneurship.

The model proposed in Figure 1 additionally incorporates the possible moderating effect of gender on the strength of the relationship between the antecedents of the TPB and EI. The specific context of a given country can shape personal beliefs and ultimately may increase the participation of women in entrepreneurship [56]. As noted in the introduction, Ecuador has two specific cultural characteristics that have a bearing in this regard: it is an efficiency-driven economy with one of the highest rates of TEA, and it also has equal gender representation in entrepreneurship. Therefore, we can expect women to perceive entrepreneurship as a socially desirable or feasible career, such that their EI may be equally influenced by the three antecedents of the TPB. Thus, the last hypotheses of the model summarized in Figure 1 are proposed:

**Hypothesis 4a** **(H4a).** *Student gender does not moderate the influence of PA on EI.*

**Hypothesis 4b** **(H4b).** *Student gender does not moderate the influence of SN on EI.*

**Hypothesis 4c** **(H4c).** *Student gender does not moderate the influence of PBC on EI.*

## 3. Materials and Methods

### 3.1. Sample and Data Collection

The study was carried out using information collected at the Universidad de Casa Grande (UCG), a private university in Guayaquil, Ecuador. Degree courses are divided among the university’s three faculties: the Faculty of Administration and Political Science, the Faculty of Communication, and the Faculty of Education. At the time of the research, the total number of students enrolled on the different courses was 1347. The questionnaire was administered to undergraduate students on different degree courses over a period of two months: August and September. For this purpose, a stratified non-probability sampling technique was used at the level of each faculty in order to obtain a sample that reliably reflected the reality of the university. A total of 770 individuals participated in the study, and the breakdown by gender was representative of the student population in Ecuador [17]. After a process of pre-evaluation and filtering of results [57] to correct for non-response bias (percentage of missing values above 15%), outliers and skewness, and kurtosis with values greater than 1, this number was reduced to 740 valid responses, covering 54.19% of the total study population and exceeding the minimum sample size for a confidence level of 95% and a margin of error of less than 5%. Therefore, given the size of the university population of the UCG (Table 1), the valid responses received provide a reliable representation of the current state of the matter at the UCG.

### 3.2. Measurement Instrument

A quantitative research design was applied for this study, and the objective at all times has been to ensure the functionality of the questionnaire. A first draft was designed on the advice of two independent entrepreneurship experts in order to validate the contents of the questionnaire. It was then tested on a small sample (*n* = 40), and minor adaptations were made to ensure it is properly tailored to the Ecuadorian context. As for the structure of the questionnaire, it is divided into four sections. The first part of the questionnaire covers the sociodemographic characteristics of the respondent (age, gender, stage of studies, university career, professional experience, and family business). The second part of the questionnaire focuses on measuring EI (five items) [13] and its antecedents, including PA (nine items), SN (two items), and PBC (six items). The third part focuses on OS and includes four items: entrepreneurial education and training; university policies and incentives; organizational culture; and networks [43,58]. The last part centres on IB and seeks to measure students′ perceptions of the costs and benefits of being an entrepreneur in relation to the institutional environment that supports (to a greater or lesser extent) the development of entrepreneurial activity. This block consists of four topics—financial barriers, market barriers, knowledge barriers [50], and government regulation [59]—represented by four items. The items are classified on a five-point Likert scale, with 1 indicating completely disagree and 5 completely agree.

### 3.3. Data Analysis

Given the complexity of the resulting structural model, it was considered appropriate to use Smart PLS (version 3.2.8) to perform the Partial Least Squares-Structural Equation Modelling (PLS-SEM) analysis [60] and present the results of the multigroup analysis (MGA). In the present study, the MGA is carried out using PLS-SEM, because it is an effective technique for evaluating moderation, allowing structural relationships to be tested one at a time [61,62]. To assess the conceptual model using PLS-SEM in relation to gender, the different measurement models are first assessed with respect to the reliability and validity of the reflective constructs, as well as multicollinearity and the relevance and significance of the weights for the formative constructs [63]. The structural model is then assessed through the R2, path coefficients, and Standarized Root Mean-Square (SRMR) values to ensure it is an appropriate approximate model for PLS-SEM [64]. After assessing the measurement and structural models, two non-parametric approaches, namely Henseler′s MGA [65,66] and the permutation test [67], are used for the MGA to study the possible moderating effect of gender (male and female). In addition, before carrying out the MGA, the measurement invariance of composite models (MICOM) is used to establish the measurement invariance between the subsets of data.

## 4. Results

### 4.1. Descriptive Statistics

Regarding the profile of the respondents, Table 2 shows that the majority are women: 64.6%, compared to 35.4% who are men. By age, students are divided into three groups: most are under 21 years old (55.0%), the second largest group comprises those between 21–23 years old (33.6%), while the rest are over 23 years old (11.3%). Students with previous professional experience represent a slight majority (51.3%) over the students without any such experience (48.7%). Regarding the stage of their degree, 38.2% of respondents are starting their degree, 39.4% are in an intermediate stage, and 22.3% are finishing their studies. In terms of their chosen degree, respondents are divided between studies in Business Administration and Management (28.5%), Communication Sciences (51.2%), Education Sciences (9.5%), and Political Sciences (10.8%).

Overall, it can be seen that the UCG students have a high EI (Table 3), registering an average value of 3.78 (SD = 1.234). In fact, more than a third (37.87%) have an EI score above 4. The mean scores for the TPB antecedents are 4.354 (SD = 0.877) for PA, considered as highly positive; this is followed by a mean value of 4.152 (SD = 0.869) for PBC, which is considered very positive; then comes SN with a score of 2.182 (SD = 1.458), which is considered low. OS registers a high score with a mean value of 4.020 (SD = 0.985), while IB shows a relatively low value at 2.464 (SD = 1.282).

Then, in order to validate the model using PLS-SEM, a three-step method is applied: assessment of the measurement model, assessment of the structural model, and MGA.

### 4.2. Assessment of the Measurement Model

#### 4.2.1. Assessment of the Measurement Model with Mode A Composites

In the first stage of the analysis, the measurement models are evaluated to ensure their validity [68]. To that end, the reflective measurement models are assessed, which entails verifying the reliability and validity of the different constructs [64]. To confirm the reliability of the latent variables (LVs) of the model, the individual reliability of each of the indicators is checked as well as the reliability or internal consistency of each of the corresponding constructs. On the other hand, the analysis of validity involves two stages: convergent validity and discriminant validity [61,69]. To determine the reliability and validity of the model, the relationship in the construct is identified; more specifically, the relationship between the empirically observable indicators and the corresponding LV. For Mode A composites, the composite reliability (CR) and the average variance extracted AVE are calculated [61].

The measurement model used in this study includes four reflective constructs: EI, PA, SN, and PBC. To evaluate the reliability of the model, factor loadings are calculated for the reflective scales, and each factor loading is compared against a cut-off value. Generally speaking, factor loadings above 0.7 are considered acceptable [61]. In this case, all the factor loadings of the individual items are above 0.7 for each of the LVs. On the other hand, construct reliability determines whether the items used to measure a construct have similar scores [61,69]. For this purpose, composite reliability (CR) (Table 3) is used as the most appropriate measure [67]. Generally speaking, values above 0.6 are considered an acceptable indication of reliability [70]; this threshold is exceeded in all cases. Regarding the assessment of the convergent validity in each of the measurement models (both overall and the subgroups), the average variance extracted (AVE) of the LVs must be above 0.5 for it to be acceptable [61,69]. Table 4 shows that the AVE of each of the constructs in each of the measurement models is above the cut-off of 0.5.

The discriminant validity indicates the extent to which each LV is different from other constructs in the model [68]. The Fornell–Larcker criterion examines the amount of variance that a construct captures from its indicators relative to the amount of variance it shares with other constructs [61,69]. This study also employs another criterion that performs better: the heterotrait–monotrait ratio (HTMT). It represents the relationship between the correlations among indicators that measure the same construct and correlations among indicators of different constructs that measure different phenomena [64]. To confirm discriminant validity, the values obtained must be below the HTMT 85 ratio. As can be seen in Table 5, each of the measurement models shows acceptable discriminant validity, in terms of both the Fornell–Larcker criterion and the HTMT 85 ratio.

#### 4.2.2. Assessment of the Measurement Models with Mode B Composites

The measurement model in this study includes two formative constructs: OS and IB. To assess the formative constructs, the multicollinearity among the potential indicators is analysed. To that end, the variance inflation factor (VIF) is used, with the results shown in Table 6 [71]. All the VIF values are below the limit of 5, indicating the absence of multicollinearity and bias in the application of the method [72]. With respect to the weights in the application of the bootstrap procedure (10,000 sub-samples), in the case of OS, all of these contribute positively to the corresponding constructs. Conversely, in the case of IB, item IB2 does not positively contribute to the model. Nevertheless, adopting a flexible approach [57,73], all items are retained in the IB formative construct.

### 4.3. Estimation and Assessment of the Structural Model

In the second stage of the analysis, the structural model for the sample of university students is assessed. The starting point for assessing the structural model is to analyse the overall goodness of fit of the model [64], which can confirm the accuracy of the global measurement model fit. To do so, the SRMR is calculated, applying a bootstrap procedure (the resampling technique yields 10,000 sub-samples), which indicates whether or not the model is well specified. The result is acceptable, since the SRMR = 0.095, and a model is considered to have a good fit when the SRMR is <0.10 [74].

After confirming the goodness of fit, the next step is to identify possible problems of multicollinearity among the variables that are antecedents of each of the endogenous constructs. In this respect, according to [75], when the VIF value is ≤3, it indicates the presence of multicollinearity.

Regarding the predictive power of the model, the coefficient of determination R2 for PA, SN, PBC, and EI is 0.104, 0.003, 0.063, and 0.218, respectively; thus, the five constructs (PA, SN, PBC, OS, and IB) account for 21.8% of the variance in EI. This value can be considered relatively acceptable for studies relating to human behaviour in general [68] and, in particular, in line with behavioural studies focused on entrepreneurship [76]. Thus, taking into account the R2 value, the results are acceptable in terms of predictive power [69].

### 4.4. Evaluation of the Hypotheses of the Main Model

The path coefficients can be interpreted as standardized regression coefficients [68]. Table 7 shows the coefficients of the different hypotheses proposed for the structural model, using a bootstrap procedure with 10,000 resamples [77]. The first set of hypotheses are all supported except for H1b (*p*-value = 0.066). Thus, PA and PBC have a positive effect on EI, but SN does not. Regarding the second set of hypotheses (H2a and H2b), as shown in Table 7, OS has a positive effect on PA and PBC. Finally, regarding the set of hypotheses H3a–H3c, addressing the relationship between IB and the different antecedents of EI, the results do not indicate that IB has an effect on the antecedents of EI.

**Figure 2:**
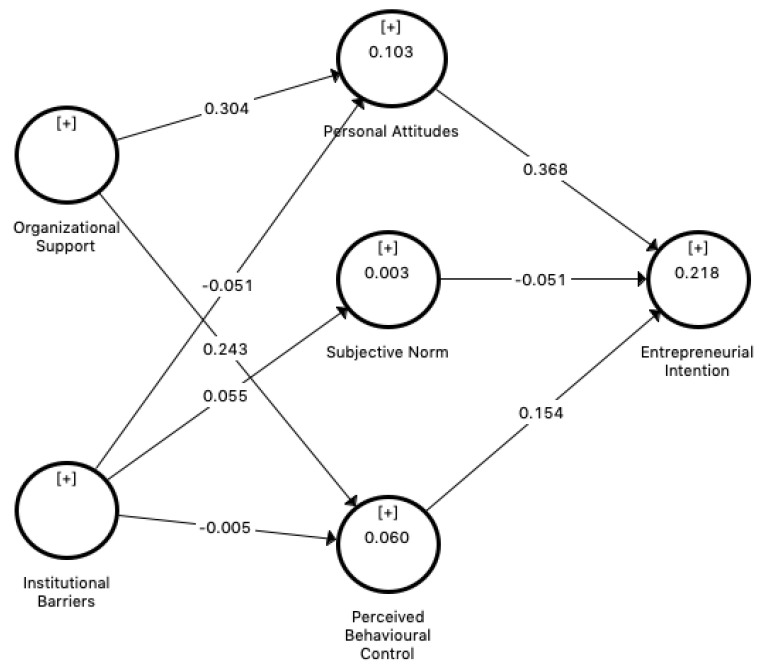
Path model with standardized factor loadings.

Therefore, almost all of the proposed hypotheses (Figure 2) are supported, suggesting that in the Ecuadorian context, the support offered by the university institution plays an important role in shaping the antecedents of students′ entrepreneurial outlook. However, the same cannot be said about the perception of barriers in the institutional environment at the national level, which is not found to have a relevant effect on the antecedents of EI.

### 4.5. Moderating Effect of Gender

Table 8 shows the results of the structural model and the test for the set of hypotheses (H4a–H4c) that assess the effect of gender on the relationships between the TPB antecedents (PA, SN, and PBC) and the dependent construct EI, using a bootstrap with 10,000 resamples and 5000 permutations. The results show that PA has a positive and significant effect on EI for male students (*p*-value = 0.000) and, to a lesser extent, so does PBC (*p*-value = 0.080). On the other hand, non-significant effects were found for male students′ SN (*p*-value = 0.434). For women, the results show a significant and positive effect of PA (*p*-value = 0.000) and PBC (*p*-value = 0.000) on EI. Furthermore, SN is found to have a significant negative effect on female students′ perception of EI (*p*-value = 0.026).

#### Multigroup Analysis (MGA)

According to Hair Jr, Hult, Ringle, and Sarstedt [68], the measurement invariance of composite models should be tested before carrying out an MGA between two or more groups using PLS-SEM. In this regard, Henseler, Ringle, Christian, and Sarstedt [64] identify MICOM as the most appropriate method for a composite model such as PLS-SEM. MICOM is a three-step procedure that consists of determining the following elements: (a) configural invariance, (b) compositional invariance, and (c) the equality of mean values and variances. In this case, MICOM is used to check that the differences between the two groups of students are due to the grouping criterion (gender) and not to potential differences that may exist in the measurement models. The MICOM procedure reveals the presence of partial measurement invariance in the two groups (men and women) (Table 8), which is necessary for the subsequent interpretation of differences at the MGA level for the PLS-SEM results [64].

Table 9 shows the results of the MGA using the two non-parametric approaches available, which are considered the most reliable and conservative tests recommended for PLS-SEM: Henseler′s MGA [78] and the permutation-based procedure [79].

The application of these two tests—Henseler′s MGA and the permutation-based method—indicates the lack of significant differences between men and women in the effect of PA, SN, and PBC on EI. Therefore, the results support hypotheses H4a–H4c. Both MGA methods used in this study thus indicate that there are no significant differences between the two genders in the university context.

## 5. Discussion

The analysis of university students’ EI in Ecuador revealed three main findings. First, of the three antecedents of the TPB, only PA and PBC showed a clear direct influence on EI. While PA was the primary predictor of EI, SN did not turn out to be significant. This finding is important, as it is in line with previous research; indeed, SN is the element of the TPB that has traditionally yielded the most contradictory evidence [15]. Both Moriano, Gorgievski, Laguna, Stephan, and Zarafshani [14] and Lortie and Castogiovanni [10] have claimed as much, pointing to studies that show a direct relationship between SN and EI [32], other studies that do not find such a relationship or find it to be barely significant [13], and a third group that shows that SN has an indirect impact on EI through PA and PBC [15,23]. In the Ecuadorian context, SN was only shown to have a direct influence on PBC. This result partially supports those from other economic contexts classified as efficiency-driven [15,23], where SN was found to have a direct effect on both PBC and PA and, particularly, on the latter. The fact that SN was not found to have an influence on PA in Ecuadorian university students could be due to the obvious cultural differences between the countries analysed; Ecuador is notable for its pro-entrepreneurship cultural values [5,16], which suggests that increasing the level of acceptance in individuals′ environment would not necessarily improve their attitudes and intentions.

Secondly, following previous studies such as those of Feola, Vesci, Botti, and Parente [24] and Urban and Chantson [23], the study examined the effects of IB and OS as exogenous antecedents of EI, exerting an influence through the components of the TPB. In this respect, students′ perceptions of the existence of IB did not show significant effects, that is, despite Ecuador not having a particularly favourable governmental climate [16], this environment did not turn out to affect any of the elements of the TPB. This could be due to the fact that the Ecuadorian population has no expectations about the elimination of such barriers and the positive view of entrepreneurship that is rooted in the culture holds sway [5,16]. Conversely, OS did turn out to affect the students′ EI through their PA and PBC. This result contrasts with that reported by Urban and Chantson [23] for academic endeavours but aligns with those found by Souitaris et al. [80] and Clarysse, Tartari, and Salter [47]. Consequently, OS could play an essential role in improving students′ EI in efficiency-driven economies.

Finally, regarding the variable gender, the MGA revealed that it does not exert any moderating influence on the elements of the TPB in a cultural context notable for gender equality in the rates of entrepreneurship. This finding particularly stands out, as the moderating effect of the variable gender has been extensively documented in the field of EI [15,52,53].

## 6. Conclusions and Final Remarks

Since fostering entrepreneurial initiatives can boost a country’s economic and social development, the study of EI and its antecedents is of particular importance. In this respect, the present analysis is based on the study of individual behaviour and the influence of its context. The main objectives were twofold: to examine the influence of the three TPB antecedents on EI in the context of an efficiency-driven economy and to study the indirect effects of IB and OS on that intention. In addition, the study examined whether the variable gender exerts any moderating effects in an economy in which there are virtually no differences in entrepreneurship rates between men and women. To that end, an empirical model of the TPB was tested and expanded by incorporating the abovementioned exogenous factors, with the analysis focusing on the level of EI in future graduates of a private Ecuadorian university.

### 6.1. Theoretical Implications

Confirming nearly all our initial expectations, the study reveals differences between the effects of TPB antecedents on university students’ EI. Moreover, the results show that the organizational context, specifically the actions of the university, shapes all students′ (men and women) behaviour towards EI equally.

The fact that the valuation of entrepreneurship as a professional opportunity is rooted in strong cultural values [16] underscores the need for a better understanding of the complexity of the antecedents of EI [23]. In terms of theoretical implications, the finding that gender does not moderate (either positively or negatively) the strength of the relationship between various antecedents and EI suggests that the role traditionally attributed to women in relation to EI may be a function of the context.

### 6.2. Policy Implications

In terms of empirical implications, the existence of IB does not appear to have any negative effects on EI. Nevertheless, it may still be advisable for policy-makers to address this aspect. The design of measures to tackle such barriers may help to boost the confidence of an already entrepreneurial population, as well as shape future perceptions. In turn, it could lead to the eventual creation of new companies in the university field. Moreover, a new law on entrepreneurship and innovation has recently been introduced in the country (February 2020) [81]; this represents an opportunity, given that the text covers financing options and seeks to simplify administrative procedures. In light of the results presented here, future modifications could be considered aimed at incorporating and communicating specific actions to remove barriers faced by new graduates. Furthermore, the finding that the OS offered at university level exerts its influence on EI through PA and PBC suggests that it is an antecedent of EI worth developing. In this regard, previous studies such as those by Saeed et al. [82] underline the importance for public policy implementation of assessing students′ perception of the support received. Through the indirect impact on students’ EI, such support could help improve their future business performance. Lastly, the fact that gender is not found to exert any moderating effects does not necessarily mean that there is no gender gap between men and women in terms of business type, performance, or opportunities. Rather, it represents an opportunity for the Ecuadorian government to address inequalities from an advantageous position in order to strengthen the role of women as key players in the country′s development process [83].

### 6.3. Limitations and Future Research

This paper is not free from limitations, which open the door to future research. With regard to the applicability of the model, the results were acceptable but limited [61,69]. In terms of the predictive power of the model, it was 0.218 for total variance in EI, which may indicate a need for caution when transferring theoretical models established in developed countries to other types of economies. Therefore, it could be worth validating the results presented here by additional studies within the Ecuadorian context; for example, focusing on other regions, students taking other degrees, or public universities. Second, the measurement of SN was based on a small number of items; in future studies, it would be advisable to include more questions that validate this indicator. Third, this is an exploratory study focused on a particular efficiency-driven economy; however, previous studies have noted the important influence of cultural differences on intention [84]. Accordingly, though it may be difficult, future studies should attempt to incorporate cultural values into TPB-based analyses in order to make comparisons between factor-driven, efficiency-driven, and developed economies. Fourth, the possibility of generalizing the findings presented here is also limited, as they relate to the population of a single university; the analysis could thus be further developed by extending it to other centres. Finally, as Feola, Vesci, Botti, and Parente [24] point out, there is a need for in-depth studies on how the various types of support are implemented by the different university institutions, since this support can vary notably from one university to another.

## Figures and Tables

**Table 1:** Sample calculation.

Degrees	Population	Minimum Sample e = 5%	Sample Obtained
Business Administration	365	198	264
Communication Sciences	696	295	307
Education Sciences	131	71	90
Political Science	155	62	79
Total N. Students	1347	626	740

**Table 2:** Profile of respondents.

Characteristics	Frequency		Percentage (%)	
	Men	Women	Men	Women
Age (years)				
<21	117	244	51.09	57.14
21–23	81	140	35.37	32.79
>23	31	43	13.54	10.07
Stage of degree				
Start	112	167	43.41	35.38
Intermediate	95	193	36.82	40.89
End	51	112	19.77	23.73
Professional experience				
Has professional experience	134	240	51.94	50.63
No professional experience	124	234	48.06	49.37
Degree				
Business Administration	67	141	25.97	29.87
Communication Sciences	146	228	56.59	48.31
Education Sciences	16	53	6.20	11.23
Political Science	29	50	11.24	10.59
Parents′ entrepreneurial background				
Yes	180	313	70.87	67.89
No	74	148	29.13	32.11

**Table 3:** Descriptive statistics.

	Mean	Std. Deviation	Percentage				
			≤1	>1−≤2	>2−≤3	>3−≤4	>4−≤5
EI	3.758	1.234	6.95	8.80	22.47	23.98	37.87
PA	4.354	0.877	1.37	2.30	11.99	27.85	56.48
SN	2.182	1.458	50.82	13.77	16.03	5.14	14.25
PBC	4.152	0.869	0.84	3.06	17.01	38.04	41.05
IB	2.464	1.282	30.27	23.78	23.95	13.26	8.73
OS	4.020	0.985	2.21	3.80	22.85	32.00	32.00

Entrepreneurial intention (EI); Personal attitude (PA); Subjective norm (SN); Personal Behavioural Control (PBC); Institutional barriers (IB); Organizational support (OS).

**Table 4:** Analysis of the measurement model.

Constructs /Associated Items	Loadings	CR	AVE
**Entrepreneurial intention (EI)**		0.841	0.677
I am very interested in creating my own company (EI1)	0.838		
I have been preparing to start my own company (EI2)	0.807		
I will make great efforts to start my own company (EI3)	0.876		
I will probably create my own company soon (EI4)	0.767		
**Personal Attitude (PA)**		0.906	0.573
I enjoy taking on personal challenges (PA1)	0.780		
I like taking on challenges from which I can learn a lot (PA2)	0.827		
I enjoy being able to solve a difficult task or problem (PA3)	0.732		
I like the idea of being my own boss (PA4)	0.704		
I enjoy taking on challenges that go beyond what I can easily do now (PA5)	0.810		
I enjoy challenging and difficult tasks through which I can gain new skills (PA6)	0.749		
I feel fulfilled when I can choose my own tasks or activities (PA7)	0.732		
I prefer to work in situations that require a high level of skills and talent (PA8)	0.741		
I often look for opportunities to develop new skills and knowledge (PA9)	0.731		
**Subjective Norm (SN)**		0.846	0.865
My close relatives like the idea of me creating my own business (SN1)	0.914		
My close friends think I should start my own business (SN2)	0.946		
**Perceived Behavioural Control (PBC)**		0.825	0.532
I diligently dedicate myself to taking a project forward (PBC1)	0.735		
I observe and try to understand where in my environment there are opportunities to solve unmet needs (PBC2)	0.747		
I face up to difficulties (PBC3)	0.711		
I am inclined to take moderate risks (PBC4)	0.730		
I dedicate as many hours as necessary to do a good job or take a project forward (PBC5)	0.721		
I′m always on the lookout for the best ways to solve the problems around me (PBC6)	0.733		

All loadings of the reflective measurement model are significant at 1% based on a two-tailed test [*t* (0.01; 10,000) = 2.577].

**Table 5:** Assessment of discriminant validity.

Fornell-Larcker Criterion					Discriminant Validity (HTMT)				
Constructs	EI	PA	SN	PBC	Constructs	EI	PA	SN	PBC
EI	0.823				EI				
PA	0.446	0.757			PA	0.506			
SN	−0.059	−0.047	0.927		SN	0.066	0.059		
PBC	0.332	0.492	0.060	0.730	PBC	0.396	0.567	0.073	

**Table 6:** Assessment of the measurement model: formative constructs.

	VIF	Outer Weight		Outer Loading	
		Original Sample (O)	*p* Values	Original Sample (O)	*p* Values
**Organizational Support (OS)**					
Technical advice on starting a business (OS1)	1.294	0.393	0.005	0.746	0
Cutting down on the formalities for starting a business (OS2)	1.384	0.240	0.068	0.672	0
Courses offered by the university on generating and developing business ideas (OS3)	1.976	0.238	0.186	0.801	0
Events on innovation and entrepreneurship held at the university (OS4)	1.864	0.419	0.004	0.845	0
**Institutional Barriers (IB)**					
Economic situation of the country (IB1)	1.399	0.494	0.184	0.832	0.020
Excessive competition in my business sector of interest (IB2)	1.221	−0.217	0.615	−0.583	0.185
Too much tax (IB3)	1.558	0.528	0.228	0.838	0.002
Too much red tape to set up a business (IB4)	1.314	0.042	0.896	0.469	0.075

**Table 7:** Direct, indirect, and total effects on entrepreneurial intent.

Hypothesis	Relationships	Path Coefficients
H1a	PA→EI	0.368 *
H1b	SN→EI	−0.051
H1c	PBC→EI	0.154 *
H2a	OS→PA	0.304 *
H2b	OS→PBC	0.243 *
H3a	IB→PA	−0.051
H3b	IB→SN	0.055
H3c	IB→PBC	−0.005

*p < 0.01.

**Table 8:** Results of invariance measurement testing using permutation.

Constructs	Configural Invariance	Compositional Invariance (Correlation = 1)		Partial Measurement Established	Equal Mean Assessment			Equal Variance Assessment			Measurement Invariance Established
		C = 1	Confidence Interval		Differences	Confidence Interval	Equal	Differences	Confidence Interval	Equal	
EI	Yes	0.999	[0.999, 1]	Yes	0.155	[−0.153, 0.149]	No	−0.166	[−0.256, 0.238]	Yes	Partial
PA	Yes	0.999	[0.999, 1]	Yes	−0.045	[−0.147, 0.148]	Yes	0.404	[−0.451, 0.417]	Yes	Total
SN	Yes	0.999	[0.931, 1]	Yes	0.057	[−0.153, 0.150]	Yes	−0.218	[−0.193, 0.181]	No	Partial
PBC	Yes	0.998	[0.996, 1]	Yes	−0.039	[−0.150, 0.149]	Yes	0.185	[−0.299, 0.282]	Yes	Total
OS	Yes	0.822	[0.899, 1]	Yes	−0.285	[−0.157, 0.150]	No	0.356	[−0.258, 0.254]	No	Partial
IB	Yes	0.820	[0.346, 1]	Yes	0.141	[−0.152, 0.155]	Yes	0.171	[−0.238, 0.227]	Yes	Total

**Table 9:** Multigroup analysis by gender.

**Hypothesis**	**Relationship**	**Path Coefficients**				**Confidence Interval (95%)**	
		**Men**		**Women**		**Men**	**Women**
H4a	PA→EI	0.402	**	0.367	**	[0.244, 0.540]	[0.283, 0.440]
H4b	SN→EI	−0.020		−0.081	*	[−0.082, 0.104]	[−0.152, −0.011]
H4c	PBC→EI	0.117		0.184	**	[−0.027, 0.233]	[0.100, 0.263]
	**Path Coefficient Difference**			***p*-Value Difference (One-Tailed)**			**Supported**
				**Henseler’s-MGA**		**Permutation Test**	
H4a	0.038			0.346		0.158	**No/No**
H4b	0.071			0.152		0.115	**No/No**
H4c	0.074			0.788		0.143	**No/No**

*p < 0.05, ** *p* < 0.01.
